# Molecular Evolution and Genetic Analysis of the Major Capsid Protein VP1 of Duck Hepatitis A Viruses: Implications for Antigenic Stability

**DOI:** 10.1371/journal.pone.0132982

**Published:** 2015-07-14

**Authors:** Xiuli Ma, Zizhang Sheng, Bing Huang, Lihong Qi, Yufeng Li, Kexiang Yu, Cunxia Liu, Zhuoming Qin, Dan Wang, Minxun Song, Feng Li

**Affiliations:** 1 Institute of Poultry Science, Shandong Academy of Agricultural Sciences, No. 1 Jiaoxiao road, Jinan, Shandong, 250023, China; 2 Department of Biochemistry and Molecular Biophysics, Columbia University, New York, NY, 10027, United States of America; 3 Department of Biology and Microbiology, South Dakota State University, Brookings, SD, 57007, United States of America; 4 Department of Veterinary and Biomedical Sciences, South Dakota State University, Brookings, SD, 57007, United States of America; Lady Davis Institute for Medical Research, CANADA

## Abstract

The duck hepatitis A virus (DHAV), a member of the family *Picornaviridae*, is the major cause of outbreaks with high mortality rates in young ducklings. It has three distinctive serotypes and among them, serotypes 1 (DHAV-1) and 3 (DHAV-3) were recognized in China. To investigate evolutionary and antigenic properties of the major capsid protein VP1 of these two serotypes, a primary target of neutralizing antibodies, we determined the VP1 coding sequences of 19 DHAV-1 (spanning 2000-2012) and 11 DHAV-3 isolates (spanning 2008-2014) associated with disease outbreaks. By bioinformatics analysis of VP1 sequences of these isolates and other DHAV strains reported previously, we demonstrated that DHAV-1 viruses evolved into two genetic lineages, while DHAV-3 viruses exhibited three distinct lineages. The rate of nucleotide substitution for DHAV-1 VP1 genes was estimated to be 5.57 x 10^-4^ per site per year, which was about one-third times slower than that for DHAV-3 VP1 genes. The population dynamics analysis showed an upward trend for infection of DHAV-1 viruses over time with little change observed for DHAV-3 viruses. Antigenic study of representative DHAV-1 and DHAV-3 strains covering all observed major lineages revealed no detectable changes in viral neutralization properties within the serotype, despite the lack of cross-neutralization between serotypes 1 and 3 strains. Structural analysis identified VP1 mutations in DHAV-1 and DHAV-3 viruses that underpin the observed antigenic phenotypes. Results of our experiments described here shall give novel insights into evolution and antigenicity of duck picornaviruses.

## Introduction

Duck hepatitis A virus (DHAV) belongs to the genus *Avihepatovirus* in the *Picornaviridae* family. As the only species in its genus, DHAV consists of three distinctive serotypes, designated DHAV-1, DHAV-2 and DHAV-3 [1]. This group of viruses can cause an acute, contagious, fatal liver disease of young ducklings (<6 weeks of age) with morbidity and mortality rates approaching 100% and 80%, respectively [2]. Among the three serotypes, DHAV-1 is the most widespread and virulent serotype that has the most significant impact on the worldwide poultry industry, while DHAV-2 and DHAV-3 have only been reported in the Taiwan and the USA and the southeastern Asia [3,4]. These two serotypes have recently become prevalent in China, Taiwan, and Korea [5–9].

In China, DHAV-1 was first isolated in 1980 in a region with a high-density poultry population in the northern part of China. The virus then quickly spread throughout all major poultry production regions of China and it has caused significant clinical problems in local poultry industry [10]. In 2013, a live attenuated virus vaccine is the only licensed vaccine against DHAV-1 in China. After implementation of DHAV-1 vaccination, DHAV-1 has become endemic with approximately 50% of the poultry flocks being infected annually. What triggers recurrent DHAV-1 outbreaks is unclear. There is little information about genetic and antigenic variations associated with DHAV-1 since its first recognition in 1980.

DHAV-3 virus was first isolated in China in 1999 [11]. Presently, both DHAV-1 and DHAV-3 viruses are co-circulating in duck farms in China and existence of both serotypes makes disease prevention and control more challenging [12,13]. There are no licensed vaccines available against DHAV-3 serotype.

As with other picornaviruses, DHAV is a single-stranded, positive-sense RNA virus. The viral genome is about 7.7kb long, packaged by a protein capsid. The capsid consists of four different structural proteins (VP1-4) and the assembled virion structure has 60 copies of capsid protein; VP1, VP2, and VP3 proteins are exposed on the surface, while VP4 protein is entirely internal. Structural and antigenic studies have shown that VP1 and to some extent VP3 or VP2, are the target for neutralizing antibodies [14,15]. Genetic changes in these regions frequently alter viral antigenic properties, which make viruses escape immunological recognition by neutralizing antibodies and as a result lead to the failure of picornavirus vaccines [16,17].

The major objective of this study was to study molecular evolution and genetic variation of the major capsid protein VP1 of DHAV-1 viruses circulating in poultry farms in China over the past 15 years since 2000. We also expanded this study into DHAV-3 viruses that have emerged as a concern for local poultry industry. Toward this goal, we determined the full VP1 sequences of 19 DHAV-1 (spanning 2000–2012) and 11 DHAV-3 isolates (spanning 2008–2014) and conducted a detailed genetic analysis including evolutionary rate and population dynamics. Our analysis also included additional 20 DHAV-3 strains whose VP1 sequences were reported previously. We focused on the VP1 protein because it is a major antigen protein that frequently undergoes genetic changes due to the evolutionary pressure from host immune system and is the principle target of neutralizing antibodies. In addition to this genetic evolution study, we also employed a cross-neutralization assay coupled with representative 4 DHAV-1 and 2 DHAV-3 strains and corresponding antisera to address antigenic evolution of duck picornaviruses. The potential implications of these experimental results are discussed.

## Materials and Methods

### Ethics Statement

Generation of chicken antisera against different DHV-1 and DHAV-3 viruses was performed at Shandong Poultry Institute, China, which was approved by the Institutional Animal Care and Use Committee of Shandong Poultry Institute (approval number 12–014). Experiments were performed under biosafety level 2+ conditions. Chickens were not sacrificed in this study. The IACUC protocol also included virus production experiments through SPF chicken embryonic eggs. Only invasive procedure used in this experiment was the inoculation of virus into eggs by an allanotic route. On the day of harvest that occurred by day 15 since the beginning of egg hatching, all live chicken embryos were euthanized by placing them at 4°C overnight. These above procedures including the embryo euthanization were discussed in the IACUC protocol.

All samples were collected from dead ducklings of infected poultry farms located in Shandong, Guangdong, Henan, and Jiangsu provinces. This work was approved by several government institutions, which included Shandong animal health and epidemiology center, Guangdong animal health and epidemiology center, Henan animal health and epidemiology center, and Jiangsu animal health and epidemiology center. None of these samples were collected from farms involving endangered or protected species.

### Virus isolation and RNA extraction

Clinical samples were collected from 19 poultry farms that experienced DHAV-1 disease outbreaks from 2000 to 2012. Poultry farms are located in four different provinces. This information was summarized in Table 1. Similarly, clinical samples were obtained from 11 poultry farms where infection and shedding of DHAV-3 viruses were documented (Table 2). For these studies, liver tissues from the diseased ducks were homogenized in sterile phosphate-buffered saline (PBS, pH 7.2) to form a 20% suspension(w/v). After centrifugation at 8000 x g for 30 min, the supernatants were filtered by 0.2μm-pore-size syringe-driven filters. The filtered suspensions were then inoculated into 11-day-old healthy DHAV antibody-negative duck embryonic eggs via allantoic cavity. Embryonic eggs were examined daily, and the allantoic fluids of dead embryonic eggs were collected following one more passage. The viral RNAs were extracted from the allantoic fluids with the MiniBEST viral RNA Extraction kit(TaKaRa) according to manufacture’s instruction.

**Table 1:** Summary of DHAV-1 strains used in this study.

Strain name	Year of isolation	Location	Serotype	Genotype	Genbank No.	Reference
1-FS/00	2000	Guangdong, China	DHAV-1	A	EU395438	From this study
1-YZ/02	2002	Jiangsu, China	DHAV-1	A	EF427899	From this study
1-CL/03	2003	Shandong, China	DHAV-1	A	EF427900	From this study
1-JH/04	2004	Shandong, China	DHAV-1	A	EU395436	From this study
1-ZZ/04	2004	Henan, China	DHAV-1	A	EU395439	From this study
1-JH/06	2006	Shandong, China	DHAV-1	A	EU395435	From this study
1-YN/06	2006	Shandong, China	DHAV-1	A	EU395437	From this study
1-LQ1/08	2008	Shandong, China	DHAV-1	A	KF826747	From this study
1-LQ2/08	2008	Shandong, China	DHAV-1	A	KF826748	From this study
1-LQ3/09	2009	Shandong, China	DHAV-1	A	KF826749	From this study
1-LQ4/09	2009	Shandong, China	DHAV-1	A	KF826750	From this study
1-LQ5/09	2009	Shandong, China	DHAV-1	A	KF826751	From this study
1-LQ7/09	2009	Shandong, China	DHAV-1	A	KF826752	From this study
1-LQ8/09	2009	Shandong, China	DHAV-1	A	KF826753	From this study
1-DZ/11	2011	Shandong, China	DHAV-1	A	KF826754	From this study
1-HY/11	2011	Shandong, China	DHAV-1	A	KF826755	From this study
1-CP/12	2012	Shandong, China	DHAV-1	A	KF826760	From this study
1-HCZY/12	2012	Henan, China	DHAV-1	A	KF826761	From this study
1-JNI/12	2012	Shandong, China	DHAV-1	A	KF826764	From this study

**Table 2:** Summary of DHAV-3 strains used in this study.

Strain name	Year of isolation	Location	Serotype	Genotype	Genbank No	Reference
3-G/99	1999	Fujian, China	DHAV-3	C	EU755009.1	Shi *et al*., 2009
3-GD/99	1999	Guangdong, China	DHAV-3	C	GQ122332.1	Yuan *et al*., 2010
3-MY02/00	2000	Sichuan, China	DHAV-3	C	KJ461995.1	ncbi.nlm.nih.gov/genbank
3-AP04114/03	2003	Korea	DHAV-3	C	DQ812093	Kim *et al*., 2007
3-AP04009/04	2004	Korea	DHAV-3	C	DQ256133	Kim *et al*., 2007
3-AP04203/04	2004	Korea	DHAV-3	C	DQ256134	Kim *et al*., 2007
3-C-YCZ/05	2005	Beijing, China	DHAV-3	C	FJ626672.1	ncbi.nlm.nih.gov/genbank
3-C-PSY/06	2006	Beijing, China	DHAV-3	C	FJ626670.1	ncbi.nlm.nih.gov/genbank
3-GD1/07	2007	Guangdong, China	DHAV-3	C	EU289393.1	Fan *et al*., 2011
3-C-YZC/07	2007	Beijing, China	DHAV-3	C	FJ626673.1	ncbi.nlm.nih.gov/genbank
3-C-GY/07	2007	Guangdong, China	DHAV-3	C	EU352805.2	Pan *et al*., 2009
3-B63/08	2008	Beijing, China	DHAV-3	C	EU747874.1	ncbi.nlm.nih.gov/genbank
3-FS /08	2008	Guangdong, China	DHAV-3	C	EU877916.1	He *et al*., 2009
3-C-PJK/09	2009	Sichuan, China	DHAV-3	C	KC282430.1	ncbi.nlm.nih.gov/genbank
3-NC/09	2009	Viet Nam	DHAV-3	C	JF925121.1	ncbi.nlm.nih.gov/genbank
3-DN1/09	2009	Viet Nam	DHAV-3	C	JF925120.1	ncbi.nlm.nih.gov/genbank
3-YT-BX/12	2012	Shandong, China	DHAV-3	C	KC191694.1	Xu *et al*., 2013
3-VF-3/12	2012	Shandong, China	DHAV-3	C	KC191681.1	Xu *et al*., 2013
3-CH1/12	2012	Shandong, China	DHAV-3	C	KJ461980.1	ncbi.nlm.nih.gov/genbank
3-HB131216/13	2013	Hubei, China	DHAV-3	C	KJ461990.1	ncbi.nlm.nih.gov/genbank
3-FX/08	2008	Shandong, China	DHAV-3	C	KF826745	From this study
3-JS/08	2008	Jiangsu, China	DHAV-3	C	KF826746	From this study
3-HKY/11	2011	Shandong, China	DHAV-3	C	KF826756	From this study
3-TA/11	2011	Shandong, China	DHAV-3	C	KF826757	From this study
3-FH/11	2011	Henan, China	DHAV-3	C	KF826758	From this study
3-HC/11	2011	Henan, China	DHAV-3	C	KF826759	From this study
3-JS/12	2012	Jiangsu, China	DHAV-3	C	KF826762	From this study
3-JM/12	2012	Shandong, China	DHAV-3	C	KF826763	From this study
3-JNA/13	2013	Shandong, China	DHAV-3	C	KF826765	From this study
3-/13	2013	Shandong, China	DHAV-3	C	KF826766	From this study
3-ZP/14	2014	Shandong, China	DHAV-3	C	KM267028	From this study

### Virus gene amplification and sequencing

Based on the sequence information of capsid genes of DHAV-1 and DHAV-3, corresponding sets of primers were designed to amplify and sequence VP1 genes of DHAV isolates. Primer sequences from 5’ to 3’ direction were CTCGAGGGTGATTCTAACCAGTT (forward primer) and GCGGCCGCTTCAATTTCCAGATT (reverse primer) for DHAV-1, and CTCGAGGGTGATTCCAATCAGCT (forward primer) GCGGCCGCTTCAATYTCCARAT (reverse primer) for DHAV-3. The gene sequences (VP1) of 19 DHAV-1 and 11 DHAV-3 viruses were already submitted to Genbank and their accession numbers were described in Table 1 and Table 2, respectively.

### Evolutionary and population genetics analysis

We used VP1 sequence information from our 19 DHAV-1 (spanning 2000–2012) and 11 DHAV-3 isolates (spanning 2008–2014) together with other DHAV isolates reported previously for this analysis. To estimate evolutionary rate for DHAV-1 and DHAV-3, we first detected whether there were virus sequences coming from recombination, which could affect rate estimation [18]. By using cutoff P value of 0.05, no recombination signal was detected using RDP3[19] for both subtypes. Identical sequences were removed by only keeping the earliest one. Then, we estimated the optimal nucleotide substitution model for each subtype using Mega5 [20]. HKY and HKY+Gamma nucleotide substitution models were then used to calculate divergence distances for DHAV-1 and DHAV-3 respectively. Rate estimation and time tree construction were then performed using Beast [21]. To find the optimal molecular clock model for rate estimation, we ran a test simulation for each dataset with ten million steps using a relaxed molecular clock model and a Bayesian skyline plot tree model. Previous study showed that if the distribution of coefficient of variation statistic contains a high frequency of zero, a restrictive molecular clock model should be used [22]. The analyses showed that restrict molecular clock and lognormal relaxed molecular clock models fit subtype 1 and subtype 3 datasets best respectively [18]. We then tested four coalescent population models (constant population size, Bayesian skyline plot, exponential population size and extended Bayesian skyline plot) as tree prior for tree likelihood estimation using path sampling algorithm [23]. Bayesian skyline plot was predicted to be the optimal model for both datasets. Finally, we ran a MCMC simulation of 10^7^ steps for each dataset and estimated evolutionary rate. Both simulations converged and the effective sample sizes were greater than 200 for all estimated parameters. Population size dynamics was analyzed using Tracer [21] with the first 10^6^ steps as burnin. The maximum clade credibility trees were generated using TreeAnnotator and showed in Figtree [21].

### Serum neutralization assay

To generate antisera for serum neutralization assay, we selected five representative DHAV-1 strains circulated between 2000 and 2012 (1-YZ/02, 1-CL/03, 1-FS/00, 1-ZZ/04, 3-FX/08 and 3-HC/11) for antigen preparations. Each antigen containing 10^4^ELD_50_ of DHAV-1 was inoculated respectively to two 21-day SPF chickens by the intramuscular route. At three weeks after the inoculation, all animals were boosted with the same dose and route as used in the original immunization. Bulk blood was collected on 15 days after the second immunization for preparation of sera. For each antigen, a pool of sera from two animals was used in the serological tests. Same strategy was used to generate antisera against two DHAV-3 viruses, 3-FX/08 and 3-HC/11.

To determine the cross-reactivity of DHAV-1 and DHAV-3 viruses, the heat-inactivated antisera, two-fold serially diluted, were respectively mixed with 200 ELD_50_ of each of the five DHAV-1 and two DHAV-3 strains. After the mixture incubated at 37°C for 1h, they were inoculated into 11-day-old healthy DHAV antibody-free duck embryonic eggs via allantoic cavity. Embryonic eggs were checked daily, and dead embryos were recorded up to 6 days post-inoculated. The serum neutralizing titers were determined by the minimal serum dilution that inhibited chicken embryo death. The titers were the mean values of two repeated experiments.

The antigenic relationship of viruses was determined by the following ratio: neutralizing antibody titer against the heterologous virus divided by neutralizing antibody titer against the homologous virus. Differences in the correlation values obtained by the polyclonal antiserum were evaluated according to standard criteria.

### Secondary structure modeling and comparison of VP1 proteins

The PSIPRED program [24] was used in this study to predict and compare at the secondary structure level VP1 proteins of DHAV-1 and DHAV-3 viruses, which were used in serum neutralization experiment.

## Results and Discussion

### Virus isolates and disease outbreaks

To study molecular evolution and genetic variation of the major capsid protein VP1 of DHAV-1 and DHAV-3 viruses circulated in China over the past decade, we isolated 19 DHAV-1 strains (spanning 2002–2012) and 11 DHAV-3 strains (spanning 2008–2014), each from individual farms where disease outbreaks were documented. These poultry farms that derived DHAV-1 isolates are located in four different provinces (Shandong, Jiangsu, Henan, and Guangdong) with most farms residing in Shandong (Table 1). Similarly, DHAV-3 viruses were obtained from these regions with the exception of Guangdong (Table 2). Virus isolates were derived from infected young ducklings (<6 weeks) with mortality rates reaching approximately 80% (data not shown).

### Genetic analysis of the VP1 protein

Viruses grown in duck embryonic eggs were purified and RT-PCR reactions were subsequently conducted on RNA extractions. The genomic coding sequences for the VP1 proteins of these DHAV-1 and DHAV-3 viruses were determined and used for genetic and structural analysis in this study. We first performed ClustalW alignment of the VP1 protein sequences. Our results showed that more than 94% amino acid sequence homology existed in the VP1 proteins of intra-serotype strains (DHAV-1 or DHAV-3), while only 76% identity was observed in the VP1 proteins between DHAV-1 and DHAV-3 (data not shown). Low degree of VP1 protein sequence homology likely played an integral role in discriminating DHAV viruses into distinctive serotypes.

### Phylogenetic and evolutionary rate analysis

We next sought to determine the evolutionary relations of VP1 genes in the context of their serotypes (DHAV-1 or DHAV-3). We also incorporated VP1 gene sequences of other DHAV strains reported previously into our analysis. Bayesian phylogenetic tree, divergence time and substitution rate for the VP1 genes were estimated for the two subtypes separately.

**Fig 1:**
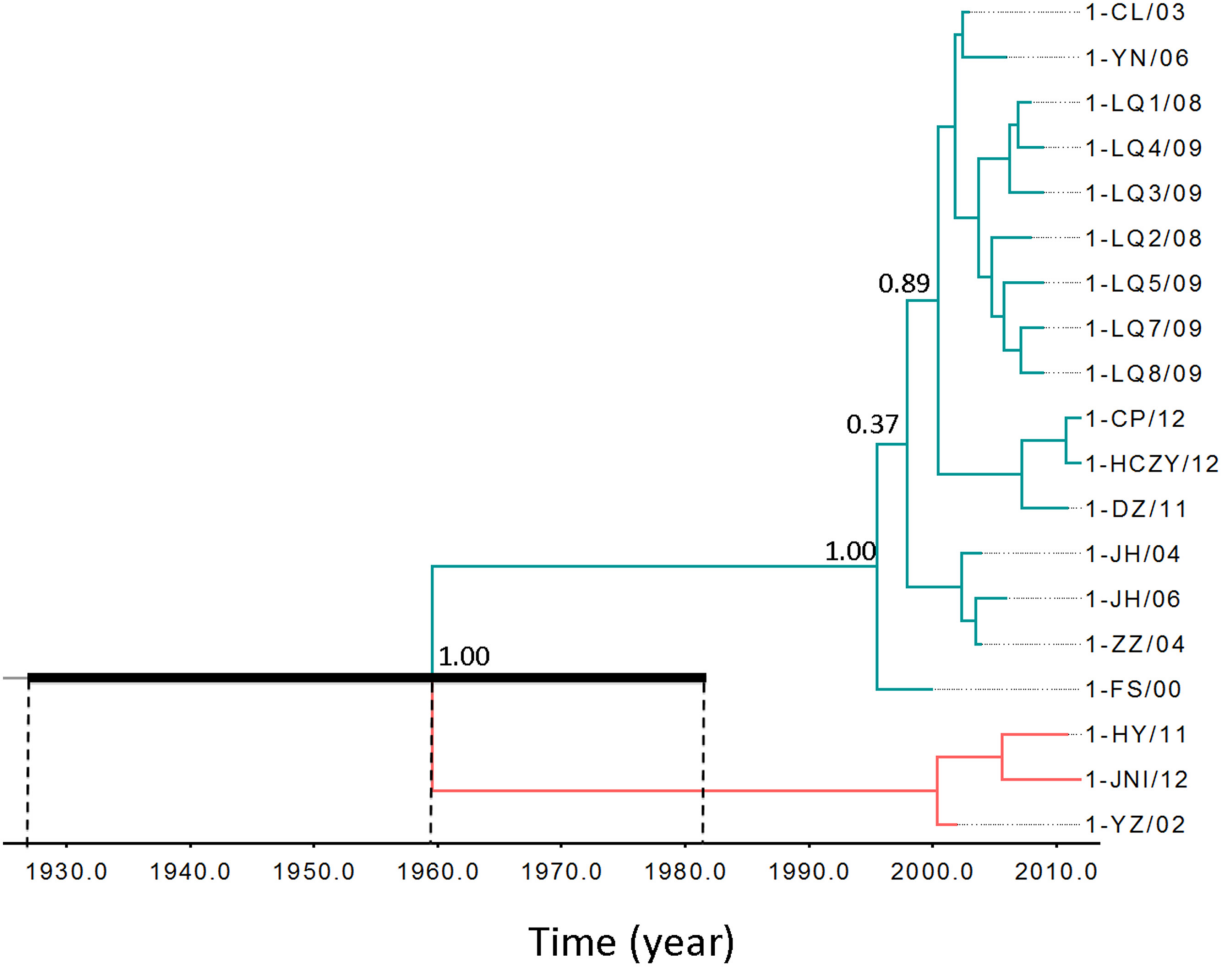
Bayesian Maximum Clade credibility tree of VP1 gene of DHAV-1 virus inferred using Beast program. The two lineages with probability support of 1 were colored green and red respectively. The probabilities of the most internal nodes were shown. The inferred divergence time was shown below the tree. The black bar represents 95% high probability interval for the most recent common ancestor. Virus strains were named in the order of serotype, abbreviations of isolation place and two digits isolation time. See materials section and Table 1 for abbreviations of isolates and their locations.

The phylogenetic tree inferred for DHAV-1 viruses shows two main lineages with Bayesian posterior probability support of 1 (Fig 1). One (indicated in green line) consisted of 16 strains that circulated from 2000 to 2012; the other (indicated in red line) included 3 strains spanning 2002–2012. The divergence time of the two lineages was traced down to 1960s, which suggests they evolved separately for a long time. This estimated time seems in a good agreement with an earliest report in 1953 that described the isolation of the first DHAV-1 virus. Because DHAV-1 was first isolated in 1980 in China, it is possible that the two lineages were brought to China separately. To further investigate whether the two lineages come from geographically isolated populations in China, we compared their geographic distributions. Probably due to the result of rapid movement of ducklings in modern poultry industry, we noticed that strains from one lineage were geographically found in place where viruses of another lineage observed. Because current analysis contains a limited number of virus strains, further exploration of DHAV-1 evolution with more strains is required.

**Fig 2:**
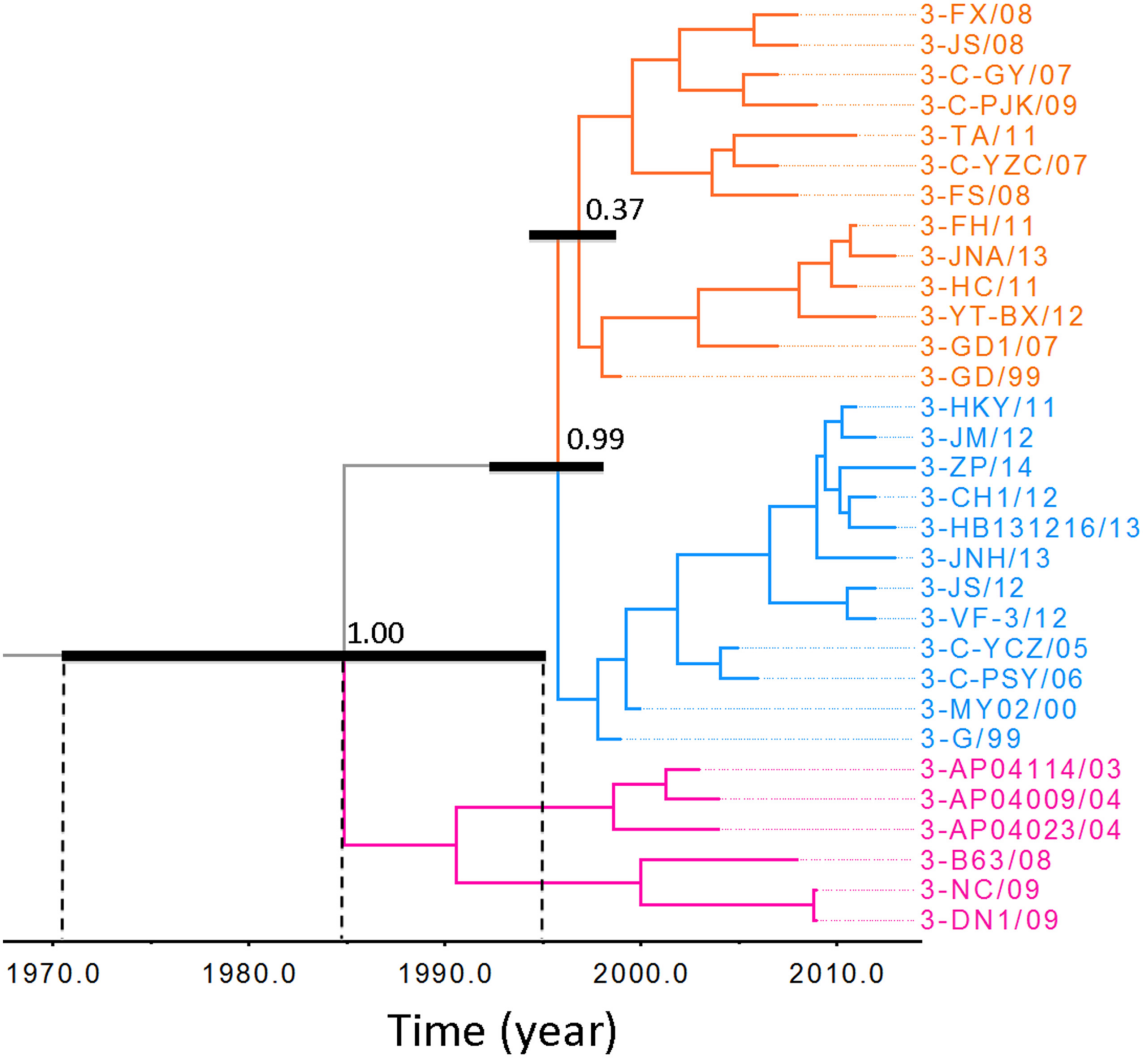
Bayesian Maximum Clade credibility tree of VP1 gene of DHAV-3 virus. The orange and blue colored branches represent one lineage while pink colored branches represents the other lineage. The probabilities of the most internal nodes were shown. The inferred divergence time was shown below the tree. The black bar represents 95% high probability interval for the most recent common ancestor. Virus strains were named in the order of serotype, abbreviations of isolation place and two digits isolation time. See materials section and Table 1 for abbreviations of isolates and their locations.

The VP1 phylogenetic tree of DHAV-3 viruses divided this group of viruses into two major lineages with Bayesian posterior probability support of 1 (Fig 2). One lineage consisted of most of our isolated strains (indicated in light yellow or blue line) covering the disease outbreaks from 1999 to 2014. It is intriguing to note that this lineage was likely further split into two sub-lineages (indicated in light yellow and blue lines respectively) with Bayesian posterior probability support of 0.99. The other lineage (indicated in pink line) contained only 6 members spanning 2003–2009. Nevertheless, additional analysis of this lineage showed that all these strains were isolated from Korea (3-AP04114/03, 3-AP04009/04, and 3-AP04023/04) and Viet Nam (3-NC/09 and 3-DN1/09) with the exception of 3-B63/08, which was isolated from a poultry farm in Beijing, China. It is likely that this lineage originated in Korea and was subsequently spread to China and Viet Nam. As such, geographical evidence for establishment of this lineage is obvious. Challenge for collecting geographical evidence in support for the presence of the other DHAV-3 lineage and two DHAV-1 lineages may be due to increased industrialization of poultry farm operation and rapid movement of ducklings within China.

We also estimated the evolutionary rates of VP1 genes of DHAV-1 and DHAV-3 viruses. Our data indicated that the estimated substitution rate for DHAV-1 and DHAV-3 viruses was about 5.57 × 10^-4^ (95% high probability density interval: 2.66x10^-4^ -8.45x10^-4^) and 1.90 × 10^-3^ (95% high probability density interval: 1.3x10^-3^ -2.6x10^-3^) nucleotides per site per year, respectively. It seemed that DHAV-3 viruses evolved about three times faster than that of DHAV-1 and is comparable to the generalized evolutionary rate of Influenza A virus [25]. The high evolutionary rate of DHAV-3 viruses may facilitate the emergence of new variants, which may warrant a regular surveillance in the field.

### Population dynamics analysis

Effective population size is usually used for studying longitudinal virus expansions over time that have been best studied in HIV[26]. We employed this analytic tool to study DHAV-1 and DHAV-3 viruses.

**Fig 3:**
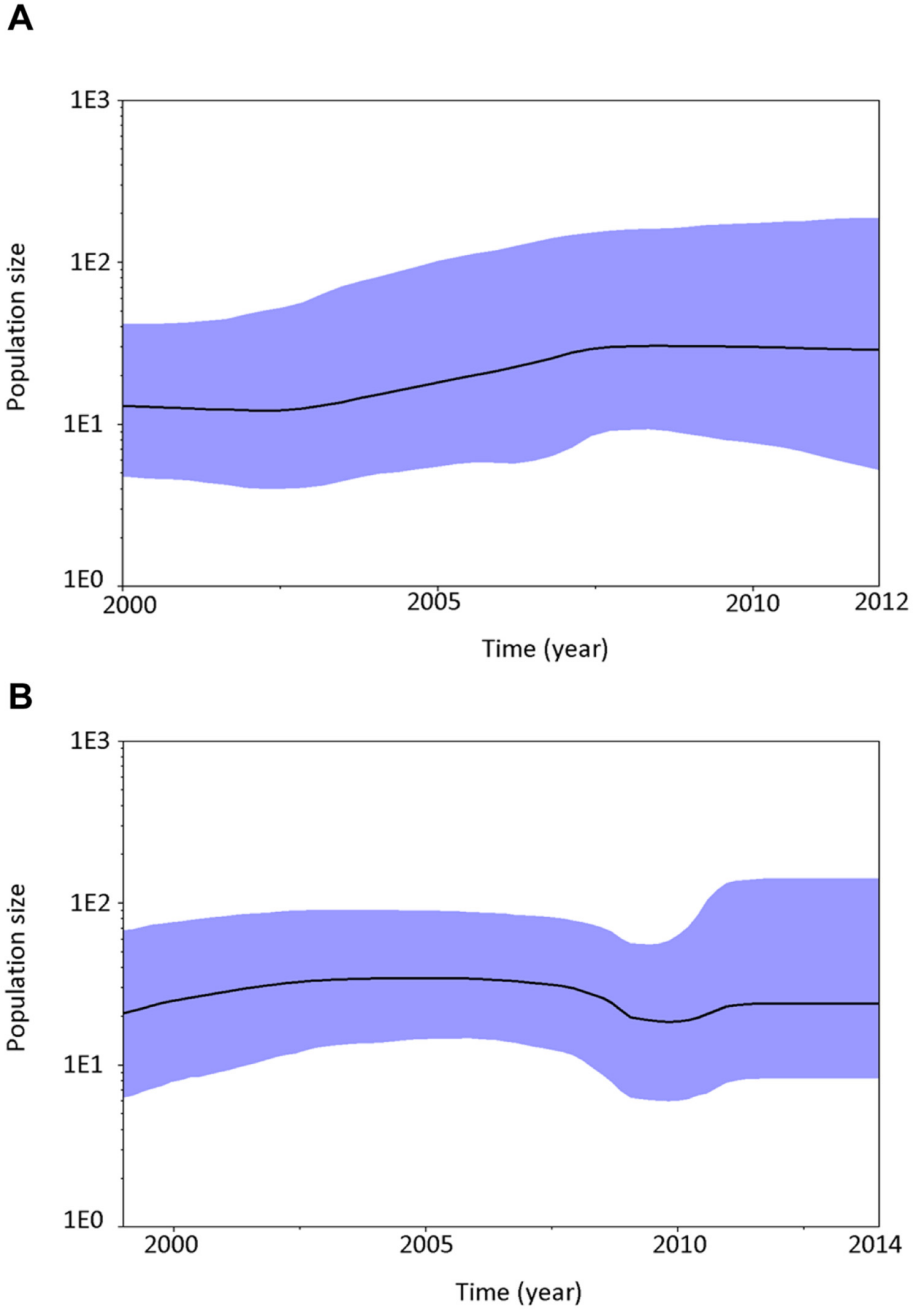
Effective population dynamics of DHAV-1 and DHAV-3 viruses. (A) Population dynamics of DHAV-1 viruses from 2000 to 2012. (B) Population dynamics of DHAV-3 viruses from 1999 to 2014. The estimated mean population size is shown using black line. At each time point, the blue colored region represents 95% high confidence intervals of population size. Because the generation time virus is unclear, the population size here is the product of effective population size and virus generation time. We assumed virus generation time is constant during evolution for our inference.

From the population dynamics plots showed in Fig 3, we observed a trend of increased infection of ducks by DHAV-1 in recent years (Fig 3A). Interestingly, there was no noticeable change in population size of DHAV-3 infection (Fig 3B), despite a little reduction in DHAV-3 viruses-associated infection landscape at 2009. It is possible that there was an evolution bottleneck around 2009 and DHAV-3 viruses may escape later by some key mutations.

### Serological characterization of the DHAV-1 and DHAV-3 viruses

The intra- and inter-serotype cross-reactivity of DHAV-1 and DHAV-3 viruses representing all major lineages or sub-lineages was measured by serum neutralization. Four sub-lineage-representative viruses and corresponding antisera for DHAV-1 (1-FS/00, 1-YZ/02, 1-CL/03, and 1-ZZ/04) and two sub-lineage-representative viruses and corresponding antisera for DHAV-3 (3-FX/08 and 3-HC/11) were used in this experiment. We also included a live-attenuated DHAV-1 vaccine (1-AV2111) and its antiserum in our study and this vaccine is currently in use against DHAV-1 infection of ducklings in China. The primary goal was to address the influence of genetic diversity on antigenic variation of these circulated viruses, especially those within the same serotype as determined in sequence analysis.

As summarized in Tables 3 and 4, five DHAV-1 viruses exhibited no detectable cross-reactivity (<10) with two DHAV-3 antisera. Similarly, two DHAV-3 viruses were insensitive to neutralization by five DHAV-1 antisera. The high cross-reactivity observed between intra-serotype viruses (DHAV-1 or DHAV-3) suggested that the observed genetic variations in VP1 over the past fourteen years for DHAV-1 or over the past six years for DHAV-3 had no effects on neutralizing properties of these viruses. We also observed that DHAV-1 vaccine-derived antiserum was capable of neutralizing all these tested DHAV-1 viruses (Table 3) and this result indicated that the DHAV-1 vaccine currently in market is protective against infection of ducklings by DHAV-1 viruses. Intriguingly, vaccine efficacy against DHAV-1 extrapolated from our serum neutralization data seemed to be in discordance with the increased population size of DHAV-1 infection over time in ducks as we estimated (Fig 3A). Several factors such as vaccination regime and coverage of duck population probably conducted in the field as a whole may cause this disagreement. This speculation was also supported by periodic outbreaks of DHAV-1 in recent years despite the implementation of DHAV-1 vaccination.

**Table 3:** Neutralization index among Different DHAV Strains.

Strains	Antisera						
	1-YZ/02*	1-CL/03*	1-FS/00*	1-ZZ/04*	AV2111*	3-FX/08*	3-HC/11*
1-YZ/02**	186	240	115	66	112	<10	<10
1-CL/03**	191	282	127	81	135	<10	<10
1-FS/00**	181	238	114	73	112	<10	<10
1-ZZ/04**	141	151	107	69	110	<10	<10
AV2111**	120	135	103	60	102	<10	<10
3-FX/08**	<10	<10	<10	<10	<10	158	112
3-HC/11**	<10	<10	<10	<10	<10	126	100

* serum

** virus

**Table 4:** Correlations Rates of Neutralization Assays testing DHAV-1 and DHAV-3 strains.

Strains	Antisera						
	1-YZ/02*	1-CL/03*	1-FS/00*	1-ZZ/04*	1-AV2111*	3-FX/08*	3-HC/11*
1-YZ/02**	1.00						
1-CL/03**	0.93	1.00					
1-FS/00**	0.99	0.97	1.00				
1-ZZ/04**	0.90	0.80	0.99	1.00			
1-AV2111**	0.84	0.80	0.99	0.97	1.00		
3-FX/08**						1.00	0.95
3-HC/11**							1.00

* serum

** virus

### Amino Acid Variability of the VP1 protein

The extremely low homology (less than 20%) of DHAV VP1 protein with its counterparts in other picornaviruses whose three-dimensional structures were resolved prevented us from conducting a structural modeling analysis. In this regard, we predicted the second structure of the VP1 protein and used it as a template for primary amino acid sequence alignment for VP1 proteins of DHAV-1 and DHAV-3 viruses analyzed in serum neutralization assay (Table 3). Our primary focus was to identify amino acid variations in the VP1 protein that discriminated viruses into serotypes 1 and 3 respectively. We used DHAV-1 strain 1-CL/03 as a reference strain to develop a secondary structure model of the VP1 protein and employed it for further analysis. Considering that DHAV-1 and DHAV-3 viruses shared 76% homology in their VP1 protein, we reasoned that the VP1 model derived from DHAV-1 should be applicable to structural analysis for the VP1 protein of DHAV-3 strains.

**Fig 4:**
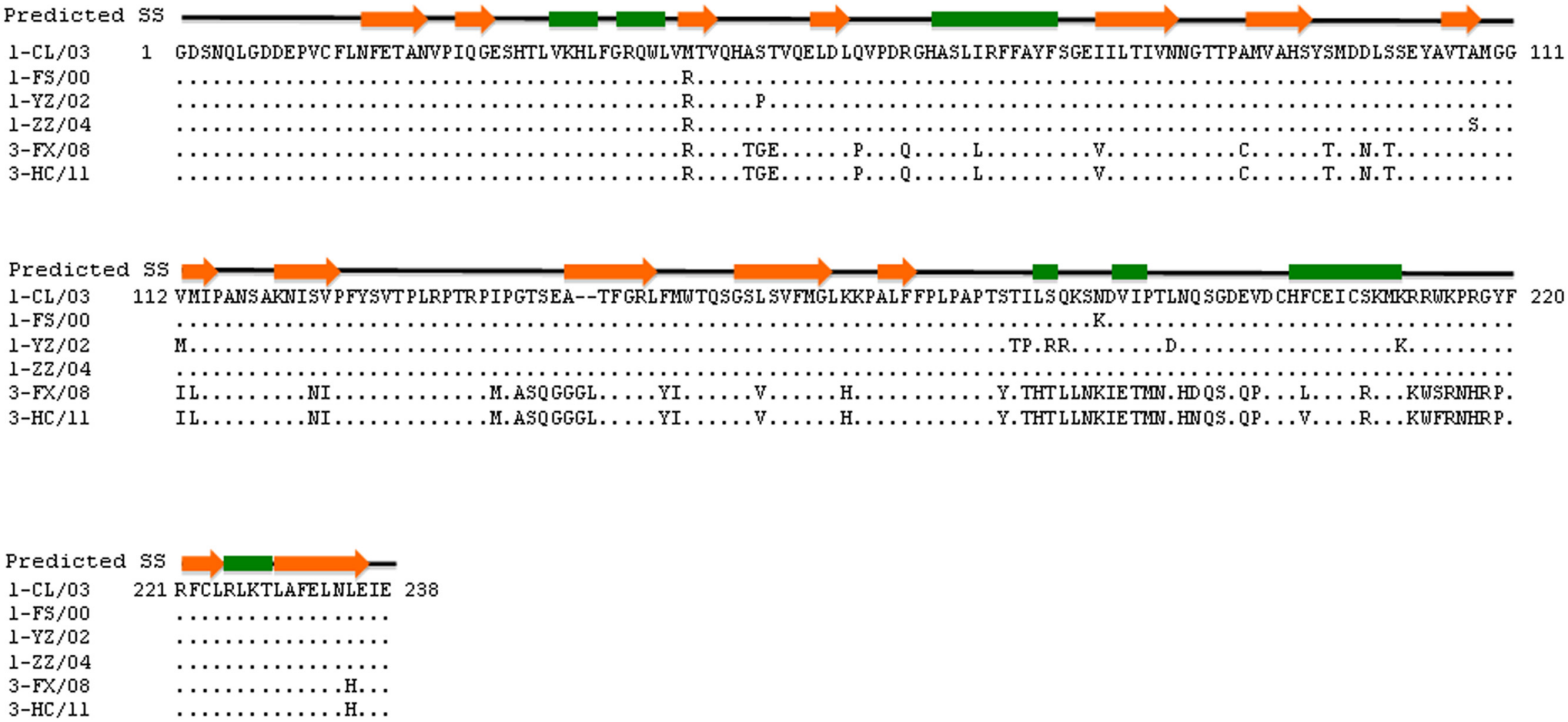
Sequence alignment and predicted secondary structure of VP1 protein of DHAV-1 and DHVA-3 viruses. Orange arrows are β strands while green rectangles are α helices. DHAV-1 strain 1-CL/03 is used here as a reference strain for development of a secondary structure model of VP1.

Secondary structure analysis revealed that VP1 protein of duck picornaviruses structurally consisted of seven α-helices, fourteen ß–sheets, and twenty linker regions (Fig 4). Despite that amino acid variations between DHAV-1 and DHAV-3 were found across the whole VP1 protein except for positions 1–47, substitutions between these two serotypes seemed to occur largely in the middle (positions 138–146) and C-terminal portions (positions 178–199 and 212–219) of the VP1 protein. These naturally occurring changes are probably responsible for the distinct antigenicity observed between DHAV-1 and DHAV-3. In addition, total thirteen substitutions occurred in VP1 proteins of three DHAV-1 viruses relative to the DHAV-1 reference strain 1-CL/03 (Fig 4). These changes located at eleven sites across the VP1 protein (positions 43, 54, 108, 112, 179, 180, 182, 183, 186, 192, and 211). These VP1 polymorphisms apparently had no effect on neutralizing properties of viruses within the serotype 1 of duck hepatitis A virus.

In conclusion, this study presents for the first time in-depth analysis of DHAV-1 and DHAV-3 viruses with a focus on VP1 gene, one of most diverse genes in the family *Picornaviridae*. Our data showed that DHAV-1 viruses are both genetically and antigenically stable over a period of the past fourteen years. Interestingly, we found that DHAV-3 viruses evolved slightly faster than DHAV-1 viruses but the increased evolutionary rate resulted in no antigenic change within this serotype. Both intra- and inter-serotype mutations identified in the VP1 protein shall provide important guidance for regular surveillance of DHAV field strains toward the identification of potential genetic mutations that may render viruses escape pre-existing immunity and cause new epidemics.
